# Covid-19 and Diabetes: A Complex Bidirectional Relationship

**DOI:** 10.3389/fendo.2020.582936

**Published:** 2020-10-08

**Authors:** Hermine Muniangi-Muhitu, Elina Akalestou, Victoria Salem, Shivani Misra, Nicholas S. Oliver, Guy A. Rutter

**Affiliations:** ^1^ Section of Cell Biology and Functional Genomics, Division of Diabetes, Endocrinology and Metabolism, Department of Metabolism, Digestion and Reproduction, Imperial College London, London, United Kingdom; ^2^ Section of Endocrinology, Division of Diabetes, Endocrinology and Metabolism, Department of Metabolism, Digestion and Reproduction, Imperial College London, London, United Kingdom; ^3^ Section of Metabolic Medicine, Division of Diabetes, Endocrinology and Metabolism, Department of Metabolism, Digestion and Reproduction, Imperial College London, London, United Kingdom; ^4^ Lee Kong Chian School of Medicine, Nan Yang Technological University, Singapore, Singapore

**Keywords:** diabetes, Covid-19, ketoacidosis, management, microangiopathy

## Abstract

Covid-19 is a recently-emerged infectious disease caused by the novel severe acute respiratory syndrome coronavirus SARS-CoV2. SARS-CoV2 differs from previous coronavirus infections (SARS and MERS) due to its high infectivity (reproduction value, R_0_, typically 2–4) and pre- or asymptomatic transmission, properties that have contributed to the current global Covid-19 pandemic. Identified risk factors for disease severity and death from SARS-Cov2 infection include older age, male sex, diabetes, obesity and hypertension. The reasons for these associations are still largely obscure. Evidence is also emerging that SARS-CoV2 infection exacerbates the underlying pathophysiology of hyperglycemia in people with diabetes. Here, we discuss potential mechanisms through which diabetes may affect the risk of more severe outcomes in Covid-19 and, additionally, how diabetic emergencies and longer term pathology may be aggravated by infection with the virus. We consider roles for the immune system, the observed phenomenon of microangiopathy in severe Covid-19 infection and the potential for direct viral toxicity on metabolically-relevant tissues including pancreatic beta cells and targets of insulin action.

## COVID-19 Pandemic 

Since its emergence in December 2019 in Wuhan, China, severe acute respiratory syndrome caused by coronavirus 2 (SARS-CoV-2), subsequently called coronavirus disease 19 (Covid-19), has ravaged the world (1) and was declared a pandemic by the World Health Organization (WHO) in March 2020 (2). As of June 15, 2020, 8,014,146 people with Covid-19 have been reported in more than 213 countries and territories, causing more than 436,005 deaths (3, 4). SARS-CoV-2 is a beta coronavirus with sequence homologies with SARS-CoV (79%) and distant similarities (50%) with Middle East respiratory syndrome coronavirus (MERS-CoV) (5, 6). It is highly contagious and transmitted between individuals through aerosolized droplets and contact with infected surfaces (7).

Data published at the beginning of the pandemic showed that Covid-19 can cause significant respiratory morbidity and mortality (8). At ~1% the mortality rate, or case fatality rate, of Covid-19 is ten times that of seasonal influenza (9–11). Age, male sex, ethnicity and existing health problems are all additional risk factors for in-hospital death from Covid-19, with age being the greatest of these: those over 80 years of age have a >500-fold higher probability of death than those under 40 (4, 12). Individuals with comorbidities are more likely to suffer a more severe disease course or die (10). The most common comorbidities with Covid-19 infection are metabolic diseases including diabetes, hypertension, obesity and cardiovascular disease (9, 10, 13). The mechanisms behind these increased risks remain unclear, and will be discussed in the present review, with a focus on the possibility that direct actions of the virus on disease-relevant tissues outside of the respiratory tract are involved.

Whereas diabetes rates in the U.K are ~4.7%, 32% of those who have died from Covid-19 had type 1 or type 2 diabetes (4). Estimates of diabetes prevalence in those who have died as a result of Covid-19 in other populations range from 20-50% (14). While obesity is associated with increased risk of a requirement for intensive care or death (15), a recent study from NHS England (16) indicates that below a body mass index (BMI) of 35 (at which point challenges are associated with adequate mechanical ventilation), increased risk is largely ascribed to altered predominance of diabetes and its complications. Similar findings were reported by Goldacre et al. (12).

In a recent study from France on ~1,300 patients (17) multivariate analysis indicated that BMI, micro- and macrovascular complications were associated with the risk of tracheal intubation or death, whereas no association was apparent for diabetes type or glycated hemoglobin (HbA1c). These apparent differences with the NHS England study may, however, reflect the fact that data on diabetes duration and HbA1c were available for ~60% of subjects in the smaller French cohort, which was also limited to patients admitted to hospital, potentially limiting the power to detect an HbA1c signal.

## Interaction Between Covid-19 and Diabetes, Hypertension, and Obesity: Epidemiological Evidence

Since the beginning of the Covid-19 outbreak much energy has been devoted to identifying risk factors for infection and severe outcomes, and understanding their underlying molecular mechanisms. Diabetes (22%) and cerebrovascular (22%) disease were identified in numerous studies as the most common distinctive comorbidities (2, 18, 19). Other retrospective studies (9–11), have revealed that the most frequent comorbidities in people infected with Covid-19 virus were hypertension (24.7%), followed by diabetes (21.2%) and coronary heart disease (8%) when these variables are assessed individually. In England, 19% of people admitted to intensive care with Covid-19 suffered from diabetes, 1/3 of whom died in hospital (4). The risk of serious complications and death from coronavirus disease in diabetic patients with diabetes in the UK population is 50% higher than that of non-diabetic people (14).

An independent association between HbA_1C_ and Covid-19 death rates was revealed in a recent cohort study based on the British population with type 1 (T1D) or type 2 (T2D) diabetes (4). This rate increased in patients with an HbA_1C_ > 58 mmol/mol, suggesting an association with hyperglycemia. Similar findings were reported by Guo and colleagues (9, 20).

Obesity is another comorbidity associated with poor outcomes after Covid-19 infection (21), and is associated with poor ventilation of the base of the lungs decreasing oxygen saturation of the blood (13). Indeed, a recent report from the National Research Center (ICNARC) (22) in the UK demonstrated that out of 196 obese patients (the majority of whom were men over 60 years old) with a BMI >35kg/m^2^ admitted to intensive care, most (3/4) needed mechanical respiration 2 h after admission. According to another study published recently in the newspaper “Le Monde”, in France, 15% of overweight or obese adults and 41% of British obese patients admitted to intensive care, were more likely to contract a SARS-CoV-2 infection and to develop severe forms (13). The main factors determining the severity of viral infection in obese patients are hormonal environment, the defective response of the innate and adaptive immune system as well as the sedentary lifestyle (4, 23). Luzi et al. (23) suggest that obesity not only increases the risk of infection and complications but also the risk of developing a more virulent viral layer, prolonging the transmission of the virus across the whole population and increasing the overall mortality rate as was the case during the H1N1 pandemic in 2009 (23). In summary, obesity and diabetes increase the duration of the disease, the requirement for intensive organ support, and increased risk of mortality.

Finally, hypertension is further, independent risk factor (though less predictive than those above) (12, 16, 24) and comorbidity in patients with Covid-19 (25).

## Angiotensin-Converting Enzyme, ACE2: Roles and Action

Multiple receptors are involved in SARS-CoV2 binding and uptake into cells, as discussed further below. The role of Angiotensin converting enzyme 2, encoded by the *ACE2* gene (26), in infection by coronaviruses including SARS (27) and SARS-Cov2 (28) is now well-established. Indeed, changes in *ACE2* expression with age and differences between sexes may contribute to the altered risks of Covid-19 infection (29). Both hypertension and diabetes are often treated with ACE inhibitors (11, 14, 30) and it has been suggested that altered ACE2 levels resulting from treatment may a contributor to disease severity in Covid-19. In results from Fang et al. (18), patients with diabetes and hypertension who had been treated with ACE inhibitors or angiotensin receptor blockers (ARB) had a high number of ACE2 receptors in the lung, and could therefore be at higher risk of developing severe symptoms, if infected with Covid-19. This hypothesis was further examined by Sardu et al. (31) in hospitalized hypertensive patients with Covid-19. Nonetheless, no firm link was established between ACEi/ARB and the prognosis of Covid-19 infection.

In normal physiology, ACE2 plays an essential role in the renin-angiotensin-aldosterone system (RAAS) (26, 32). Activation of the RAAS system takes place during a loss of blood volume, a decrease in blood pressure or when the serum concentration of Na^+^ falls, promoting in juxtaglomerular cells in the kidney to release renin. The latter cleaves angiotensinogen to release angiotensin I (1–10), which is further converted by ACE1 into angiotensin II a potent vasoconstrictor. Angiotensin II is degraded by ACE2, which cleaves angiotensin II to generate Ang (1–7).

Ang (1–7) is specific for AT1Ra (AGTRAP) and Mas receptors, which are associated with vasodilation (26, 32, 33). Therefore, viral depletion of ACE2 in SARS-CoV2 may reduce vasodilatory tone, contributing to the microangiopathy. Of note, AT1R are present on both pancreatic beta and alpha cells in mice (34) and humans (35). Hence lowered ACE2 levels, and consequently of Ang (1–7), may impact hormone secretion from islets.

Could ACE2 present on beta cells represent a direct target for virus entry, potentially leading to the dysregulation or destruction of these cells, promoting a (potentially irreversible) loss of insulin production? Low but detectable (1–4 reads per kilobase of transcript per million reads, RPKM) levels of *ACE2* mRNA are reported in purified human beta cells (35), and ACE2 immunoreactivity has also been described on these cells (36). However, the selectivity of the antibodies used in the latter study was not tested directly, and ACE2-mediated SARS-CoV2 entry into beta cells entry remains unproven.

Might elevated levels of ACE2, facilitating viral entry, contribute to increased disease risk and mortality in metabolic disease? *In vitro* and *in vivo* studies in disease settings have indicated that *ACE2* expression is increased in heart failure, systemic and pulmonary hypertension and diabetes mellitus (33). ACE2 is expressed in several tissues and organs (**Table 1**). These include endothelial cells as well as vascular smooth muscle cells (26). In the kidney, ACE2 is expressed on the apical surface on the proximal tubules and glomerulus. Its expression has also been observed in the gastrointestinal tract (42). ACE2 has also been reported in the central nervous system and in glial cells (37, 38), and SARS-CoV2 infection *via* the receptor may contribute to the loss of smell (anosmia) observed in many patients (https://covid.joinzoe.com/post/uk-anosmia-covid). Whether ACE2 is expressed by olfactory neurons remains to be demonstrated. ACE2 levels throughout the rest of the human brain appear to be low (42). Finally, thyroid tissue has been shown to express high levels of ACE2 (43). To date, there is limited information regarding the risk of SARS-CoV2 infection in patients with thyroid disease. However, some knowledge regarding an impact of SARS-CoV2 on thyroid function can be inferred from the severe acute respiratory syndrome (SARS) epidemic, when a decrease in serum levels of hormones triiodothyronine (T3) and thyroxines (T4) had been observed in infected patients (44).

**Table 1:** Summary of the various organs/tissues expressing ACE2.

Organs	Type of cells expressing ACE2	Impacts in term of risk	Type of Receptors	References
Heart	Myocytes	Cardiac failure	ACE2	Burrell et al. (26)
Brain	Glial cells and neurons	Loss of smell, CV stroke, epilepsy	ACE2 receptors present in the central nervous system (CNS)	Gupta et al. (37), Bittman et al. (38)
Liver	Biliary epithelial cells	Proteinuria	ACE2	Sun et al. (39)
Intestine	Enterocytes	/	ACE2	Ziegler et al. (40)
Lungs	Pneumocytes	Respiratory failure	ACE2	Mourad et al. (33)
Pancreas	Beta cells	Decreased insulin production	ACE2	Luzi et al. (23), Yang et al. (36)
Kidney	Nephron proximal bypass tube cell	Renal failure	ACE2	Burrell et al. (26)
Adipose tissue	Adipocytes	Severe obesity	ACE2	Shoemaker et al. (41)

The third column highlights the failures that the various organs will undergo when the virus binds to the ACE2 receptors present in the different types of cells expressing the angiotensin 2 converting enzyme.

## SARS-CoV-2 and the Immune System in Diabetes

As discussed below, infection with SARS-CoV2 appears to have actions on both immune cells and on endothelial cells pertinent to its interactions with metabolic disease (23, 45).

**Figure 1:**
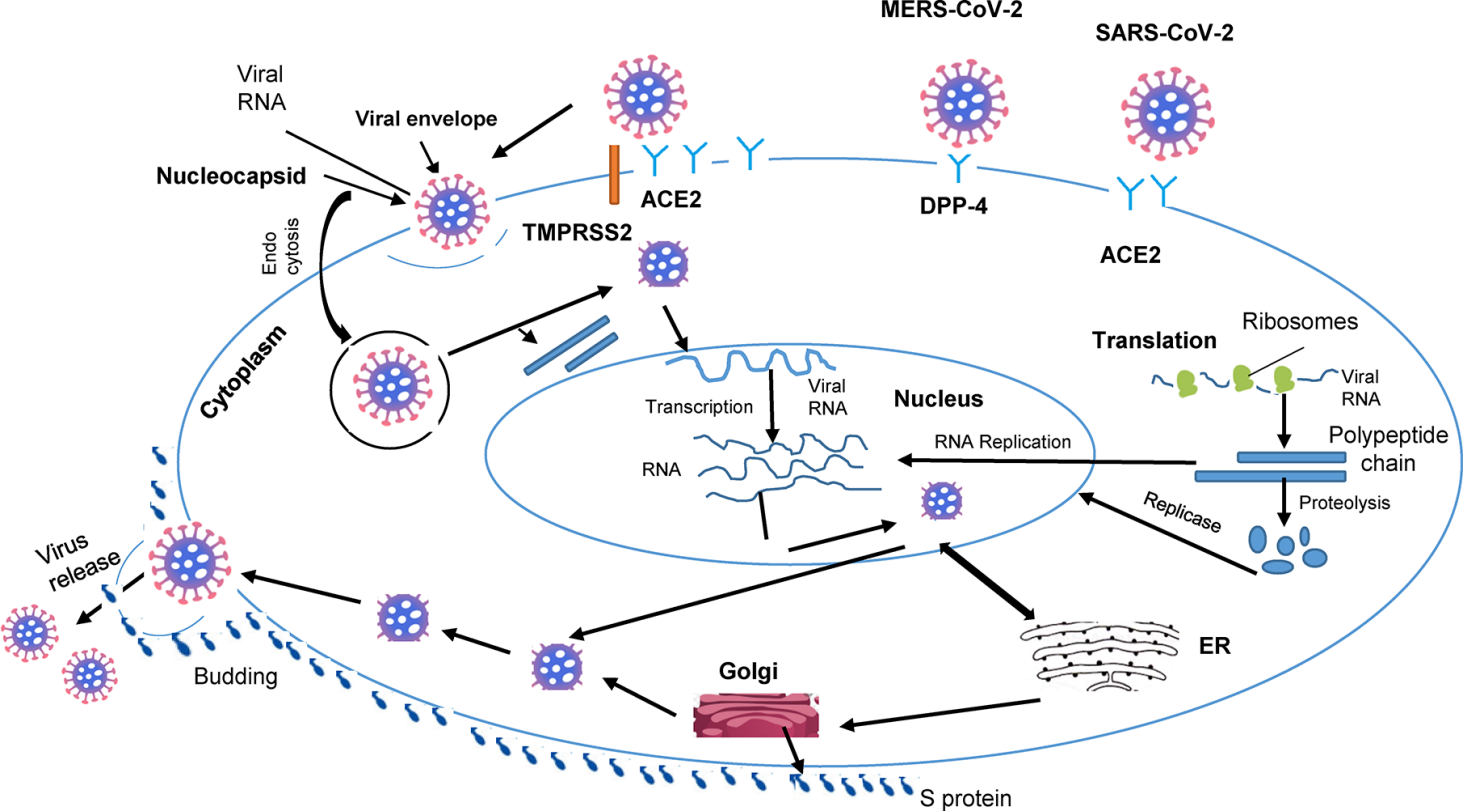
Cellular pathogenesis of coronavirus infection. The lipid envelope (viral) includes spike glycoprotein binding to receptors (ACE2) on the surface of the host. The, entry of cell by endocytosis followed by membrane fusion and release of the virion into the cytosol. Entry of viral RNA into the nucleus and production of new strands of viral RNA and viral proteins. Viral RNA output from the nucleus. Assembly, budding and release of new viruses, impact on organs.

Infection of individual cells with the virus begins with the cleavage of the Spike protein (S), a surface glycoprotein carried by the spicules, in 2 subunits S1 and S2. The S1 subunit of the Spike protein binds to the N-terminal region of ACE2 (33). The second subunit, S2, then interacts with the transmarine protease assisted with serine 2 (TMPRSS2) which cleaves Protein S to allow viral entry (46). RNA from the viral genome is then released into the cytoplasm, allowing viral replication (47) (**Figure 1**). Thereafter, the virus’ genomic RNA, together with the envelope glycoprotein and nucleocapsid protein, form vesicles containing virions *via* the cell’s secretory pathway, which go on to fuse with the plasma membrane and release the virus from the host cell (5, 6).

Cytokine production is a key element of the inflammatory and immune response to viral infection. After release from the host, the virus is first recognized by the innate immune system *via* molecular pattern recognition receptors (PRR), such as type C lectin receptors, and the Toll Like Receptor (TLR) NOD receptor. Exposure to virus causes the expression of inflammatory factors by various pathways, in particular the maturation of dendritic cells, and the synthesis of interferons whose role is to limit the spread of the virus and accelerate the phagocytosis of viral antigens (5).

An important feature of Covid-19 infection is a lowered level of lymphocytes in the blood. Implying a similar process after infection with SARS-Cov2, monocytes and T cells are infected with MERS-CoV *via* dipeptidyl peptidase 4 (DPP-4), while SARS-CoV infects primary human monocytes as well as dendritic cells (48). This could be due to destruction of these key cells, which might be a contributor to a weakened immune response, facilitating virus proliferation in Covid-19. However, it is important to bear in mind that a failure to observe lymphocytes in the blood might reflect their homing or recruitment to other infected organs such as the lung (49).

The binding of the virus to immune cells also leads to the synthesis of interleukin 6 (IL-6) which, in turn, leads to signaling both in *cis* and in *trans*. In *cis* signaling, IL-6 binds to lymphocytes *via* cognate receptors. The overproduction of these cytokines (in a “cytokine storm”) leads to the risk of multiorganic lesions (35) this might be the main cause of morbidity in subjects with Covid-19. On the other hand, in *trans* signaling, IL-6 binds to receptors on endothelial cells and may lead to hyper-coagulation, an important risk factor for pulmonary embolism and death (50). The fact that patients with severe forms of SARS-CoV-2 infection express higher than normal plasma concentrations of IL-6 (7) which prompted Mehta and colleagues (51) to propose that secondary hemaphagocytic lymphoistiocytosis (sHLH), characterized by an overproduction of cytokines and expansion of macrophages, is found in severe cases of Covid-19 since the main target cells of SARS-CoV-2 are alveolar macrophages expressing ACE2. A recent clinical trial (52) with Kevzara (Sarilumab), an IL-6 receptor antibody, has, however given mixed results, with positive effects observed in “critical” cases but negative in “severe” cases.

## Covid-19 and Thrombotic Microangiopathy

Microangiopathy involves damage to smallest blood vessels and can be the result of the formation of small blood clots, termed thrombotic microangiopathy (TMA) that contribute to renal and neuronal pathology (53). A preexisting microangiopathic disease burden (and markers thereof, e.g. poor glycaemic control and duration of diabetes) are strong risk factors for disease severity (54). TMA is reported as a frequent event in Covid-19 and is likely to involve endothelium-mediated complement activation (55). Given high levels of expression of ACE2 in endothelial cells and podocytes, activation of this process may present a unifying mechanism for viral action in a range of susceptible tissues, including kidney and heart. Clinical observations (56) suggest that SARS-CoV-2 infection facilitates the induction of endotheliitis in several organs as a direct consequence of viral infection. This could contribute markedly to life-threatening complications, such as venous thromboembolic disease and multiple organ failure. Complement inhibition (57) may thus represent an important therapeutic target. Moreover, the inflammatory effects of cytokines may also result in vascular endothelial cell injury which could result in thrombosis (58). Therefore, it is possible that the so-called Covid-19 Associated Coagulopathy (CAC) may be a result of the increased inflammatory response (59).

## Covid-19 and Diabetes: A Vicious Circle?

**Figure 2:**
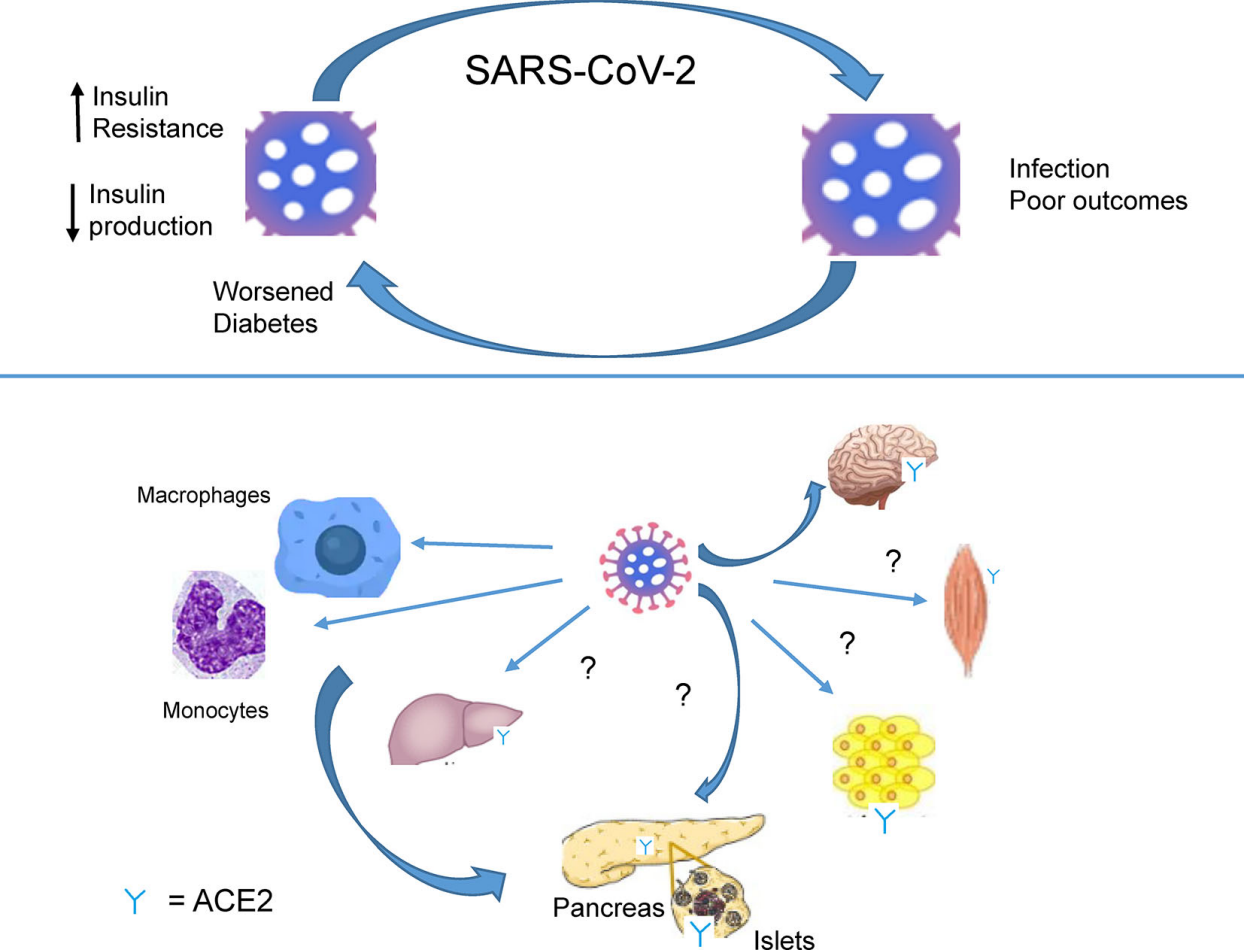
SARS-CoV-2 infection worsens diabetes. Recognition by SAR-CoV-2 of receptors present on immune cells (macrophages, monocytes.) and ACE2 receptors, expressed in several tissues (brain, muscle, adipose tissue, liver, pancreas). Infection causes lowered insulin production and insulin resistance by presently undefined mechanisms.

Emerging data indicate a bidirectional relationship between T2D and Covid-19 (**Figure 2**). Firstly, and as described above, preexisting diabetes is a risk factor for poor outcomes and death after Covid-19. Several explanations for this association are possible. Of these the impairment, at different levels, of the innate and adaptive immune response is likely to be involved in the poorer ability to fight infection in patients with diabetes, and particularly in those who are obese (19, 23, 30). People with all forms of diabetes are at increased risk due to defective innate immunity as well as adaptive immunity (14) Severe Covid-19 infection significantly reduces the numbers of natural killer cells, notably CD4^+^ and CD8^+^ cells, as well as CD4^+^ as CD8^+^ lymphocytes (7). The mechanisms behind these very recent findings are unclear: whether they involve interactions of the virus with beta cells or target tissues for insulin action, or are the result of an indirect effect of an immune response (“cytokine storm”), is unclear (**Figure 2**).

The association between Covid-19 and hyperglycemia in elderly patients with T2D (60) seems likely to reflect metabolic inflammation and exaggerated cytokine release. Strikingly, recent data suggest that SARS-CoV2 infection can lead to a deterioration in glycemic control, involving both profound insulin resistance (requiring as much as 50–100 U insulin/h) and impaired insulin secretion, together leading to diabetic ketoacidosis, DKA (61, 62). Thus, frequent cases of severe DKA have been observed on admission to hospital of patients with Covid-19 (14) and contribute to mortality and morbidity (9).

With respect to the glycemic deterioration seen in patients with preexisting T2D during Covid-19, a very recent report (63) provides the intriguing observation that *ACE2* expression at both the mRNA and protein is increased substantially in human beta cells in response to response to inflammatory cytokines, presumably rendering these cells more susceptible to infection.

## Type 2 Diabetes Management in Covid-19

The appropriate management of Covid-19 in people with T2D has been debated actively during the present pandemic, especially with regards to the range of drugs best suited for glycemic control that may also reduce the risk of infection and attenuate the severity of complications. This discussion is of great importance since early glycaemic control may be an important therapeutic option to reduce the poor outcomes in hyperglycaemic Covid-19 patients (64). As an example, it has been shown recently that Covid-19 infection management with the drug tocilizumab was not optimally achieved during hyperglycaemia in both diabetic and non-diabetic patients. Moreover, preclinical models found an indirect link between ACE2 upregulation and several anti-diabetic drugs (65–68). We discuss here presently used treatments both in the context of diabetic emergencies (such as DKA; see above) and for less acute management of hyperglycemia, where different regimens may be required. Given that a number of treatments are now re-purposed as potential therapeutics for Covid-19 (69), the anti-inflammatory action of several anti-diabetic drugs may also be explored in this light.

Insulin remains a safe choice under all circumstances and is considered the first-line treatment in hyperglycemic critically-ill patients, and may require very high doses (see above). In patients infected with Covid-19, insulin infusion has been shown to be an effective method for achieving glycemic targets as well as reducing the risk of severe symptoms, when compared to patients that did not receive an infusion (70). It has been shown to modulate inflammatory mediators, suppress toll-like receptors (TLRs) implicated in innate immune responses, and suppress pro-inflammatory transcription factors in mononuclear cells (71). Importantly, insulin reduces activation of the pro-inflammatory nuclear transcription factor κB (NF-κB), both in obese non-diabetic and critically ill patients (72, 73). Even though there is no direct link between insulin and ACE2, it has been demonstrated that, in diabetic mice, insulin treatment can attenuate a disintegrin and metalloproteinase-17 (ADAM-17) expression in the kidney.

Glucagon-Like Peptide 1 Receptor Agonists (GLP-1RAs) are an important line of pharmaceutical agents that are especially effective in obese people with T2D to address post-prandial hyperglycaemia. Like insulin, they are also exerting anti-inflammatory activity, as a growing amount of evidence suggests that they may have beneficial effects on lipid profiles and blood pressure, as well as reduced markers of systemic inflammation and improved endothelial dysfunction (74, 75). Specifically in respiratory diseases, GLP-1RAs reduced cytokine concentration and attenuated pulmonary inflammation in preclinical models of lung infection and injury (76–78). Moreover, the GLP-1RA liraglutide has been shown to downregulate immune cell infiltration and protein expression of cytokines, and markedly attenuate NF-κB activation in a chronic asthma preclinical model (79, 80).

Dipeptidyl peptidase 4 (DPP4) inhibitors are often prescribed in combination with other agents for the treatment of T2D. Unlike the majority of anti-diabetic treatments, DPP4 inhibitors do not appear to alter the immune system response in patients with or without T2D (81). However, DPP4 inhibitors have recently been associated with a better clinical outcome in patients with COVID-19 (82) potentially due to the fact that DPP4 is a predicted coronavirus receptor (83). Further details on DPP4 molecular mechanisms and clinical importance have recently been reviewed (20).

Metformin is an oral hypoglycemic agent which is widely used as first-line therapy for T2D as it suppresses hepatic glucose production and increases muscle glucose uptake (84). Similarly to insulin and GLP-1RA, metformin has been suggested to improve chronic inflammation indirectly, *via* insulin resistance and hyperglycemia improvement, but also directly by inhibiting NF-κB *via* AMP-activated protein kinase (AMPK)-dependent and independent pathways (85–88). Another suggested mechanism of the anti-inflammatory action of metformin is inhibition of advanced glycation end products (AGEs) formation, which promote inflammation and glycoxidation (89). However, metformin is not indicated for use in critically-ill hospitalized patients, especially if their hepatic function is impaired, and in cases of dehydration: lactic acidosis is a risk in these circumstances (14). Although concerns have been raised over the use of the anti-rheumatic drug chloroquine or hydroxychloroquine as a potential Covid-19 therapeutic based on studies in animals (90), these were, until recently, used safely together with metformin in humans: however, the licensing of chloroquine use in Covid-19 has recently been withdrawn by the FDA.

Thiazolidinediones are a class of T2D drugs which includes the oral agent pioglitazone. Pioglitazone improves insulin sensitivity through its action at peroxisome proliferator-activated receptor γ1 (PPARγ1) and PPARγ2, and affects lipid metabolism through action at PPARα (91). Although potentially indirect, pioglitazone has been proven to reduce monocyte gene and protein expression of cytokines in patients with impaired glucose tolerance (92). Of note, pioglitazone was found to reduce lung injury by controlling adipose inflammation in a cecal ligation puncture model in mice (93) as well as exert a direct effect on lung inflammation and fibrosis (94). As a result, it has been hypothesized that it should be considered among the drugs currently used against COVID-19 (95). However, use of this drug class in Covid-19 patients is presently very limited.

An additional class of anti-diabetic medications associated with lowering inflammation are the glifozins, or SGLT2 (sodium-glucose cotransporter-2/SLC5A2) inhibitors, which attenuate reabsorption of glucose in the kidney to lower blood sugar. So far, this reported anti-inflammatory effect focuses on the kidney, cardiovascular system and pancreas, rather than lungs (96, 97), though it has been shown to indirectly reduce pulmonary infection in diabetic mice (98). Although SGLT2 inhibitors are associated with dehydration and anorexia and therefore may pose a risk for critically ill patients, a new study focusing on hospitalized adult patients with Covid-19 commenced in April 2020, with the aim of understanding the substantial cardio- and nephroprotective effects of SLGT2 inhibitors in reducing disease progression, complications, and all-cause mortality (99). Recent U.K. guidelines (100) have, nonetheless, advised suspending the use of SGLT2 inhibitors in people with Covid-19 due to the risk of euglycaemic ketoacidosis.

It is important to note that many of the therapeutics mentioned, such as GLP-1RA and SGLT2 inhibitors, have also been strongly associated with improved cardiovascular outcomes (9, 31, 34). This may be of great importance, as growing evidence links Covid-19 with cardiovascular complications (35), in addition to respiratory disease, especially since SARS-CoV-2 can directly infect engineered human blood vessel organoids (36).

**Figure 3:**
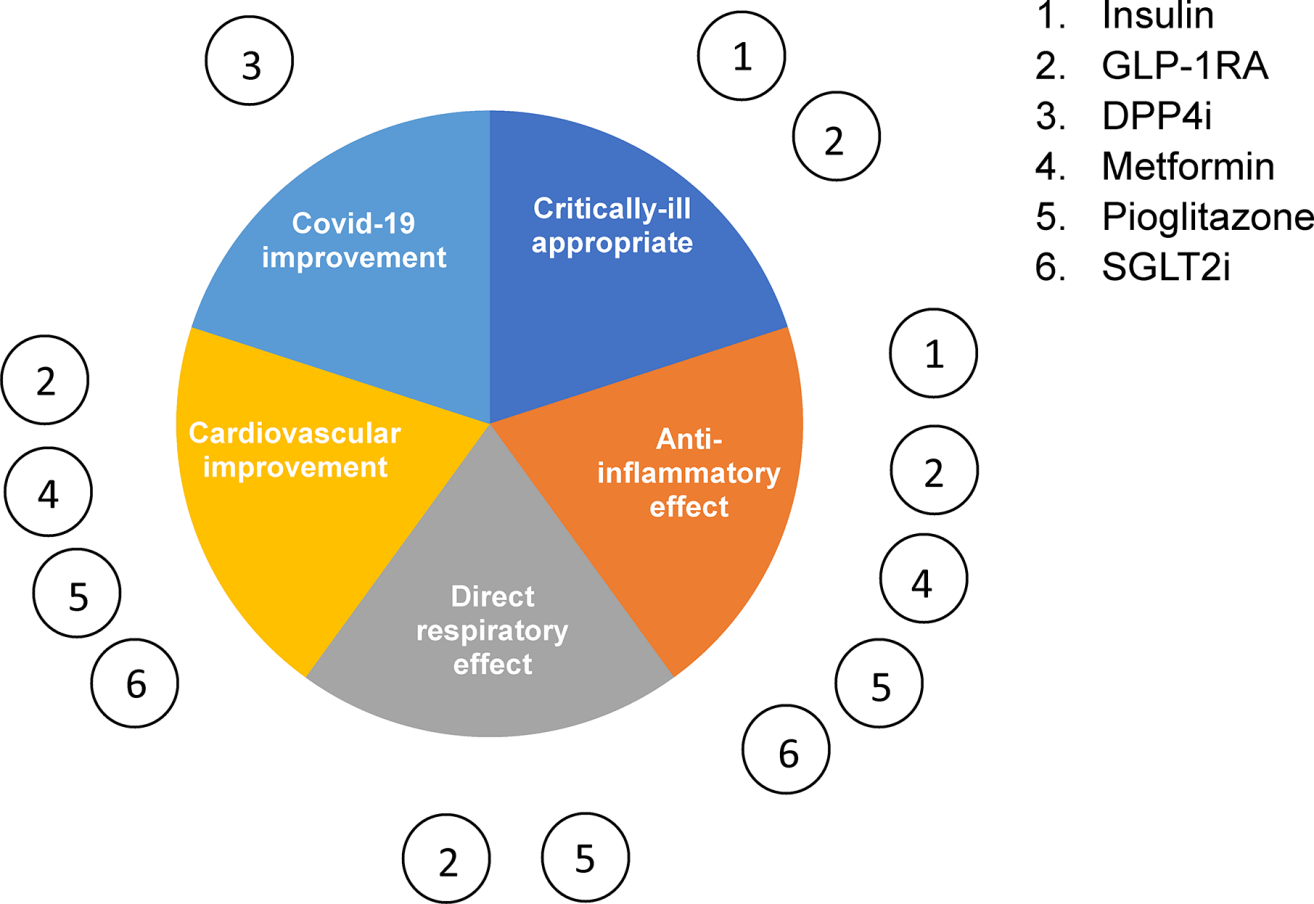
Type 2 diabetes management therapeutics in Covid-19. The six main classes of glucose-lowering drugs summarized according to their reported effect on inflammation, cardiovascular disease, respiratory disease and critically ill patient safety.

Overall, most anti-diabetic therapeutics demonstrate anti-inflammatory effects (**Figure 3**), either indirectly by improving insulin resistance or directly by down-regulating proinflammatory pathways such as those involving nuclear factor κB (NF-κB). Moreover, several drugs have been shown to act directly on the lungs and have a pulmonary effect in respiratory infection and injury. However, most of the studies reporting this utilized preclinical models. It will therefore be important to validate these findings clinically in the context of experimental coronavirus infection in order to determine which anti-diabetic treatments are optimal for a combined management of T2D and Covid-19.

## Conclusions and Perspectives

There is now considerable evidence that diabetes is both a risk factor for, and a condition worsened by, SARS-CoV-2 infection. Whereas weakened immunity, alongside impaired kidney function – both features of both aging and diabetes – likely drive the former, exaggerated immune responses probably underlie the latter. Further research will be needed to answer these closely interlinked questions, and we provide suggestions for prioritization below.

With respect to heightened susceptibility to poor Covid-19 outcomes in people with diabetes, key questions include the role of the endothelium and blood hyper-coagulation. Could this affect islet or kidney function? Most evidence suggests that levels of the relevant SARS-Cov2 receptors (ACE2 and DPP4) are low, but not zero, in these tissues - DPP4 is detectable in islets, liver and kidney - suggesting that immune cell-mediated effects are the more likely. More detailed and robust assessment of the expression at the protein level, and sub-cellular localization, of these receptors in the above tissues, and their susceptibility to SARS-Cov2 infection *in vitro* and *in vivo*, are needed.

Regarding the effects of viral infection on glucose control: there is as yet little evidence to suggest that deteriorating glycemia and metabolic control in patients with diabetes outlasts infection, suggesting that immune-mediated destruction of pancreatic beta cells akin to Type 1 diabetes is unlikely to be involved. Nevertheless, formal analysis of this question, i.e. epidemiological assessment of the reversion of glycemic symptoms post Covid-19, as well as histological quantitation *post mortem* of beta cell numbers, and islet infiltration with immune cells, are now called for. The most question remains: are the effects of the virus on glycemic control purely the result of an over-active immune system, with immune cells and inflammatory cytokines acting on multiple tissues (which)?, or are there *direct* actions of the virus on tissues and organs relevant to metabolic homoeostasis (beta cells, other islet hormone-secreting cells, liver, fat, muscle, brain, kidney etc.)? Studies in animal models overexpressing or inactivated for ACE2 (101) or other SARS-CoV2 receptors may be informative here. Where feasible, more in-depth physiological studies (e.g. glucose tolerance tests, hyperinsulimic or hyperglycemic clamps) are required in patients to assess insulin sensitivity (102) and beta cell glucose responsiveness (103) with greater precision. Follow-up studies will then be required at the cellular level to understand the molecular mechanisms behind altered beta (or other) cell function or insulin action, and to understand whether, and through what membrane trafficking pathways, viral replication and shedding occurs in these cell types. Finally, clinical trials in man, informed by the results of the above, will be needed to determine which of the existing, and potentially new, treatments, are likely to be efficacious in reducing glycemia-related medical emergencies as well as the more severe manifestations of Covid-19.

## Author Contributions

GR, HM-M, and EA prepared the figures. All authors contributed to the article and approved the submitted version.

## Funding

GR was supported by a Wellcome Trust Investigator Award (212625/Z/18/Z), MRC Programme grants (MR/R022259/1, MR/J0003042/1, MR/L020149/1) and by Diabetes UK (BDA/11/0004210, BDA/15/0005275, BDA 16/0005485) project grants. VS is the recipient of a Diabetes UK Harry Keen Clinician Scientist Fellowship. This project has received funding from the European Union’s Horizon 2020 research and innovation programme *via* the Innovative Medicines Initiative 2 Joint Undertaking under grant agreement No 115881 (RHAPSODY). This Joint Undertaking receives support from the European Union’s Horizon 2020 research and innovation programme and EFPIA.

## Conflict of Interest

GR has received research funding in the past for unrelated studies from Sun Pharma (GR is also a consultant) and from Servier. GR confirms that neither of these organisations had any involvement in the present “study” (i.e., review).

The remaining authors declare that the research was conducted in the absence of any commercial or financial relationships that could be construed as a potential conflict of interest.

## Abbreviations

ACE2, angiotensin-converting enzyme 2; DKA, diabetic ketoacidosis; SARS-CoV2, severe acute respiratory syndrome coronavirus-2; T2D, type 2 diabetes.
